# A Contribution to the Study of Joint-Bodies from within Present in Articulations Otherwise Apparently Normal

**Published:** 1915-03

**Authors:** Aime Paul Heineck

**Affiliations:** Chicago, Illinois


### TEXAS MEDICAL JOURNAL

**Dr. F. E. Daniel, Founder. Established July, 1885**

MRS. F. E. DANIEL, - Publisher and Managing Editor

**Published Monthly.—Subscription, $1.00 a Year.**

VOL. XXX AUSTIN, MARCH, 1915 No. 9

**The publisher is not responsible for views of contributors**

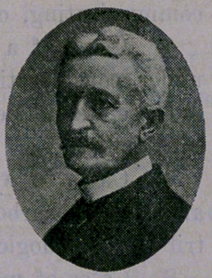

### Original Articles.

### A Contribution to the Study of Joint-Bodies From Within Present in Articulations Otherwise Apparently Normal.

BY AIME PAUL HEINECK, M. D., CHICAGO, ILLINOIS.

An analytical review of all cases of joint-bodies originally reported in the English, French and German medical literature from 1890 to 1913, inclusive.*

Our knowledge of the etiology, pathological anatomy, symptomatology, and of the various methods of treatment of joint-bodies is as yet incomplete and, therefore, calls for much further study.

The articular bodies which we herein discuss were unassociated with joint-lesions other than those determined by the presence of the joint-body or by the violence responsible for its existence.

To avoid misstatements and to have accurate data as substructure of our conclusions, we only analyzed cases in which the diagnosis of joint-body was verified at the operating, dissecting or postmortem table.

In formulating our conclusions, we excluded from consideration:

1. Cases reported with insufficient data or with only unimportant details.
2. Supposed or actual cases of fragmented, displaced or detached semilunar cartilage.
3. Supposed cases of joint-body, proven at time of operation to be extra-articular bodies.
4. Cases of extra-articular foreign bodies of intra-articular origin.

*All the English, French and German publications to be found in the John Crerar Library, Chicago, Ill., U. S. A.

5. Cases of foreign body lodged in joint-capsule diverticula communicating, or not, with the general synovial cavity.

6. Cases of a nature so distinct from that of the joint-bodies under consideration that their inclusion would serve no useful purpose, would needlessly confuse the reader (Pendulous chondra-sarcomata).

7. Cases in which a pre-existing or co-existing disease of the articulation harboring the joint-body might be considered a contributory etiological factor (gonorrheal, suppurative arthritis).

8. Cases of mono- or poly-articular arthritis deformans.

We attempt to determine some facts relative to joint-bodies originating within the organism:

a. What is their incidence—

(1) As to age?

(2) As to sex?

(3) As to articulation involved?

(4) As to association with pre-existing or co-existing, congenital or acquired, anomalies of the affected articulation?

b. Their etiology, nature and pathological anatomy.

c. Their symptomatology.

d. Their differential diagnosis.

e. Their treatment: Operative or non-operative. If operative, should one resort to local or general anesthesia? To joint-lavage? To joint-drainage? To immediate closure of the articulation? To immobilization? What is to be the nature of the post-operative treatment?

f. Results of operative treatment.

g. Conclusions.

INCIDENCE AS TO AGE.

The reader will keep in mind that the age given in case reports is not that at which the joint-body first became manifest, but is the age of the patient at time of operative relief.

The two youngest patients operated for joint-bodies originating within the organism were nine and twelve years old, respectively. The knee was the articulation involved in both cases. The oldest patients were sixty-four and sixty-five years of age, respectively. The other cases in which the age is stated admit of the following tabulation:

From 14-19 years....................45 patients.
From 20-29 years....................97 patients.
From 30-39 years....................41 patients.

From 40-49 years....................34 patients.
From 50-60 years....................10 patients.

Forty-one patients were subjected to operation for removal of elbow joint-bodies. Twenty-six of them were under twenty-one years of age.

INCIDENCE AS TO SEX.

Thirty-three of the patients were females, and the remaining two hundred and sixty-two were males.

The relative freedom of the female sex from this pathological condition applies to all articulations. Out of the two hundred and forty-five patients operated upon for knee joint-bodies, twenty-six are said to have been females. One female patient was operated for a joint-mouse in right hip.

Women, owing to occupation, mode of life, etc., are less exposed to traumatic influences than men, and, therefore, pathologic conditions due to external violence are far less frequent in females than in males.

INCIDENCE AS TO ARTICULATION INVOLVED.

The condition may be mono-articular, poly-articular, or bilateral. Dissimilar joints may be involved in the same individual.

The unequal distribution as regards articulation involved is well recognized. In some articulations, joint-mice are pathological curiosities. Thus, Morestin found in the right piso-pyramidal joint of a female dissecting-room subject, a smooth, ovoid, bean-sized, osseous, pedunculated body.

The joint-incidence in the other cases is as follows:

*Upper Extremity.*

| | | |
|---|---|---|
| Metacarpo-phalangeal joint of thumb.............. | | 1 |
| Right .................................... | 1 | |
| Radio-carpal joint ............................ | | 2 |
| Right .................................... | 1 | |
| Left ..................................... | 1 | |
| Shoulder joint ............................... | | 1 |
| Left ..................................... | 1 | |
| Elbow joint .................................. | | 41 |
| Side not mentioned........................ | 1 | |
| Right .................................... | 30 | |
| Left ..................................... | 10 | |

*Lower Extremity.*

| | | |
|---|---|---|
| Second meta-tarso-phalangeal joint |  | 1 |
| Ankle joint |  | 4 |
| Side not mentioned | 1 | |
| Right | 2 | |
| Left | 1 | |
| Hip joint |  | 3 |
| Right | 2 | |
| Left | 1 | |
| Knee joint |  | 250 |
| Side not mentioned | 42 | |
| Bilateral | 8 | |
| Right | 99 | |
| Left | 101 | |

ASSOCIATION WITH PRE-EXISTING OR CO-EXISTING, CONGENITAL OR ACQUIRED, ANOMALIES OF THE ARTICULATION WHICH HARBORS THE JOINT-BODY OR BODIES.

Primary joint-bodies occur in articulations otherwise normal, occur in articulations, the seat of other pathologic processes. In the latter condition there will be present a double disability: That determined by the presence of the joint-body and that dependent upon associated conditions. They have been found in connection with joint-bodies from without, with extra-articular foreign bodies, with dislocations, with ruptured ligaments, with fractured articular surfaces, etc.

ETIOLOGY.

In the causation of joint-bodies, external violence either of a direct nature, such as that attending bumps, blows, falls, etc., or of an indirect nature, such as forcible muscular or ligamentous contractions, energetic torsions, efforts, sprains, etc., appears to be the main etiological factor. Indirect injuries are commonly due to movements productive of abnormal pressure or great traction on osseous or cartilaginous bone ends.

Patients often do not give a history of an injury sufficiently serious to have impressed itself upon their memory. Nevertheless, in two hundred and eighteen cases, a distinct and definite history of trauma is recorded.

In the elbow cases it is stated:

In twenty-seven cases of elbow joint-body, the causative violence was:

| | |
|---|---|
| A sprain of upper extremity | 1 case. |
| A fall upon outstretched hand | 2 cases. |
| A dislocation of elbow | 2 cases. |
| Throwing of a stone, of snowballs | 2 cases. |
| Fall upon elbow | 8 cases. |
| An elbow injury | 12 cases. |

Among forms of violence productive of knee joint-bodies, we find the following:

| | |
|---|---|
| Jumping | 8 cases. |
| Blow upon knee | 10 cases. |
| Sprained knee | 16 cases. |
| Injury to knee | 46 cases. |
| Fall upon knee | 56 cases. |

In a few cases, a history of more than one injury is obtained. In such cases one, at times, finds it difficult to determine which of the two traumatisms is the causative one. In other cases, the first violence partly detaches the joint-body, the second one completely frees it from its base. A partly detached joint-body may remain in that state for a long period.

It has been amply demonstrated that trauma, slight or severe, may break off chips of articular cartilage and underlying bone without inflicting necessarily serious injury upon other portions of the joint. Slight force, attempting to avoid a fall, motion carried beyond normal limits, etc., may be the determining factor. The femoral condyles are the most frequent site of origin of traumatic knee joint-bodies.

As regards the etiology of joint-bodies, the following facts are demonstrated:

a. One or more osseous, cartilaginous or osteo-cartilaginous fragments may be forcibly detached into the knee-joint, from the femoral condyles, tibial tuberosities or patella; may be forcibly detached from any articular surface into the joint cavity, in the formation of which that surface, in part, enters.

b. Organization of blood-clot and resulting formation of a fibrous mass acting as a joint-body is a possible sequel of articular hemorrhage.

c. Lipomata originating in the subsynovial fatty connective tissue may become pedunculated and hang into the joint cavity.

d. Exceptionally, the joint-body is a free or pedunculated fibroma, enchondroma, osteoma, or enchondrosis. Purely cartilaginous joint-bodies are not common.

e. As a result of injury, a portion of synovial membrane and underlying fatty connective tissue undergoes thickening and induration. Should this portion project into the articulation, it will be nipped repeatedly during joint-movements.

f. In its normal state, the synovial membrane fringes may contain nodules of cartilage. Morbid conditions, as sprains, irritation, etc., act as a stimulus to these cartilage cells, causing them to subdivide, multiply and give rise to growths projecting into the joint cavity; if these growths are snipped off by the joint-movements, loose joint-bodies result.

In the greater number of cases only one joint-body is present; in some, two are present; in some, three; in some, four; in some, five; in some, six; in others, eight; in others, ten.

The joint-body or bodies may be free, pedicled, or only partly detached. The joint-body may be adherent to the synovial membrane, the articular capsule or the bone.

a. The joint-bodies were bound down by adhesions in nineteen cases.

b. They were pedicled in twenty-six cases.

c. They were free in eighty-six cases.

Joint-bodies vary in size, shape, and surface characterictics. They may be ovoid, oblong, flat oblong, spheroidal, pyriform, biconcave, biconvex, concave-convex, flattened, reniform, etc. They are pea-sized, lima-bean-sized, olive-sized, pigeon-egg sized, large chestnut sized, hen's egg sized, etc.

The detached body either remains free or becomes adherent to bone, articular cartilage or synovial membrane by an osseous, fibro-cartilaginous or purely fibrous bond of union.

The cases of joint-bodies which we studied may be classified, according to structural characteristics, as follows:

| | | |
|---|---|---|
| A. | Fibrous in structure | 11 cases. |
| B. | Subserous fat lumps or subserous fat swellings | 6 cases. |
| C. | Lipoma, pedicled or non-pedicled | 12 cases. |
| D. | Osteoma | 1 case. |
| E. | Enchondrosis | 1 case. |
| F. | Osseous in structure | 15 cases. |
| G. | Cartilaginous in structure | 103 cases. |
| H. | Osteo-cartilaginous in structure | 123 cases. |

The large number of osteo-cartilaginous bodies is due to the fact that, in by far the larger number of cases, a piece of underlying bone is broken off with the cartilage, the line of cleavage being in the bone and not between the bone and cartilage.

| | | |
|---|---|---|
| I. | Nature of body not manifest | 32 cases. |

Complete examination of the entire articular cartilage is rarely possible; nevertheless, in many cases one sees, during the course of the operation, the gap left by the detached joint-body. In knee cases, this defect is commonly situated on the outer surface of the internal femoral condyle in front of and close to the seat of insertion of the posterior crucial ligament.

### SYMPTOMATOLOGY.

Two sets of symptoms call for consideration: First, those referable to the injury sustained by the joint as a whole at the time of the partial or complete detachment of the joint-body; second, those directly dependent upon the joint-body itself. In many cases, the subjective and objective symptoms are so distinct that the condition is easy of recognition. In other cases the symptoms are either inconstant, mild or difficult of interpretation. Partly detached joint-bodies may persist for years as such, causing at long intervals more or less joint-irritation. Symptoms may be entirely lacking. Joint-locking may be the first symptom.

The main signs and symptoms of this condition are the following: Pain and tenderness, joint-effusion, joint-disability, crepitus, joint-locking, direct palpation of joint-body and X-ray evidence of joint-body existence.

Pain and tenderness are present in a large number of cases and may or may not be associated with other symptoms. They may be constant, intermittent or present only on attempted motion. They vary in degree, being in some cases merely a sensation of discomfort, or rheumatic in nature.

*Joint-effusion.*—The synovial membrane is fretted by the presence of the joint-body and from this and from the initial trauma an effusion results (hemorrhagic,—rarely—sero-hemorrhagic or serous). The effusion contributes, directly and indirectly, to impairment of joint-function. When massive and of long duration, it over-distends the joint-ligaments, and there results a pathological laxity of these structures which allows abnormal joint-movements (lateral movements in knee), impairs joint-stability and predisposes to luxation.

*Joint-disability.*—Joint-bodies are mechanical obstacles to movement, which may or may not produce great pain. Joint-disability is reported as marked impairment of function in ninety-three cases, as joint-weakness in thirty-four cases, and as limitation of motion in fifty-one cases.

*Crepitus.*—Crepitus is a sensation and a sound; one feels and hears crepitus. In eighty-seven of the studied cases it is said to have been distinctly elicited. In all cases in which the authors report the presence of crepitus, the joint-body or bodies present were cartilaginous, osseous or osteo-cartilaginous in nature.

*Joint-locking.*—Sudden attacks of acute severe, excruciating pain associated with joint-locking, constitute one of the most pronounced symptoms of this condition. It occurs irrespective of the nature of the joint-body. The pain is so severe that not infrequently the patient is nauseated or faints. In the knee, joint-locking usually manifests itself as follows: While walking, patient has a very sudden acute pain; he is compelled to stop, to sit down, the joint-movement is not completed, the joint is locked, usually in the partly extended position of the articulation. Often the patient knows where the body is impacted.

*Direct Palpation of Joint-body.*—Should the clinician, in examining the affected articulation, palpate one or more joint-bodies, the diagnosis is clinched. Palpation informs the surgeon as to size, number, surface characteristics and degree of mobility of joint-mice. In one hundred and seventy-five cases one or more joint-bodies were palpable before operation.

In the elbow, the joint-body may be felt between the ulna and radius or between the radius and humerus or ulna and humerus.

Inability to palpate a joint-body does not exclude its existence.

A palpable joint-body may be immovable, slightly movable or very movable. It may be movable in all directions or only in one direction. A movable body may lose its mobility and later recover it.

One of the most valuable agents for the diagnosis of joint-bodies is Roentgenography. Its value, according to our experience, is dependent to a great degree upon the ability of the radiographist to take and interpret negatives. The X-ray examination should include both an antero-posterior and a lateral view. Joint-bodies from within containing no calcific deposits or no osseous foci, will not cast a shadow upon the X-ray plate.

*Differential Diagnosis.*—In the differential diagnosis of this condition the difficulties encountered are due to various reasons:

a. The causative violence may not have been sufficiently severe to leave a lasting impression upon the patient's memory.

b. The condition may not come under the surgeon's observation in the early stage; therefore, one, at times, is in doubt as to whether an existent joint-body is primary in nature or secondary to a pre-existing or co-existing joint-affection.

c. In the absence of an exploratory incision, the interior of a joint cannot be inspected.

d. Radiography may fail to make the case clear.

With few exceptions, the mistakes made in reference to the diagnosing of this condition, are avoidable. Joint-bodies located in deep-seated joints (hip, etc.) are the ones which puzzle the clinician. As to the symptom joint-effusion, we should bear in mind that a hydrops which resists appropriate treatment is not a disease sui generis. A joint-effusion calls for interpretation. Is it primary? Is it secondary? If secondary, secondary to what? To the presence of a joint-body? The palpatory findings are all important. The detection of a palpable joint-body, movable or immovable, makes the diagnosis self-evident. Joint-locking occurs in only one other common condition: Partial or complete detachment of one or the other, almost always the internal, semilunar cartilage. In the latter condition, the pain is most marked over the interarticular line. In the differential diagnosis between a joint-body and a torn or detached semilunar cartilage, Roentgenography is an invaluable aid. It helps to diagnose the presence, number and location of joint-bodies. The semilunar cartilages consist of a core of fibrous tissue arranged transversely and longitudinally, with a covering above and below of white fibrous cartilage. As they contain neither calcific nor ossific deposits, they are not opaque to the X-rays, and when torn or displaced cartilages give no findings on the X-ray plate. Osseous and osteo-cartilaginous joint-bodies appear as shadows on the X-ray plate.

A partly or completely detached semilunar cartilage calls for an arthrotomy as a preliminary step to its proper fixation or removal. All primary, free or pedicled, joint-bodies call for an arthrotomy as a preliminary step to their operative removal. Since both conditions call for opening of the synovial cavity, failure to differentiate one from the other, is neither a disastrous, not even a very significant mistake.*

---

Secretary Rawlings of the Relief Association made an appeal for medical inspection in the public schools before the doctors' Luncheon Club at the Metropolitan Hotel at Fort Worth. He advocates the maintenance of an inspection staff and staff of nurses to care for deficient children.

*This article will be continued in April number of the Texas Medical Journal. Dr. Heineck giving the treatments for joint-bodies.

